# Bioactive compounds from *Holothuria atra* of Indian ocean

**DOI:** 10.1186/2193-1801-3-673

**Published:** 2014-11-14

**Authors:** Devaraj Isaac Dhinakaran, Aaron Premnath Lipton

**Affiliations:** Centre for Marine Science and Technology, Manonmaniam Sundaranar University, Rajakkamangalam, Kanyakumari District, Tamil Nadu 629502 India; Marine Biotechnology Laboratory, Central Marine Fisheries Research Institute, Vizhinjam, 695521 Thiruvananthapuram, Kerala India

**Keywords:** *Holothuria atra*, Bioactive compounds, Cell lines Herpes simplex virus MCF-7

## Abstract

The sea cucumber (*Holothuria atra*) extracts have been evaluated for the presence of bioactive compounds and various biological activities. The methanol extracts showed anti proliferative activities against the Hela and MCF-7 cell lines. Similarly the inhibitory effects of Herpes simplex virus 1 and 2 cells were detected using the plaque reduction assay. The extracts of *H. atra* were purified using the silica gel column chromatography. The active fractions collected were observed for antimicrobial activity. The GC-MS analysis showed the availability of 59 compounds. The active bioactive compounds found in the *H. atra* were analyzed and their structure was identified using the ^1^HNMR and ^13^C NMR experiments.

**Electronic supplementary material:**

The online version of this article (doi:10.1186/2193-1801-3-673) contains supplementary material, which is available to authorized users.

## Introduction

The marine holothurians are spiny skinned invertebrates, which form important commercial group among the echinoderms. In India, about 200 species of holothurians are known of which 75 species are from shallow waters within 20 m depths (Cuvillier 2002). (James 1994) suggests that great potential exists for the extraction of valuable bioactive compounds from the sea cucumbers in the Indian coast. A new immunomodulatory lead Cumaside a complex of monosulfated triterpene glycosides from the sea cucumber *Cucumaria japonica* possesses cytotoxic activity against Ehrlich carcinoma cells (Aminin *et al.*^2004^). Sphingoid base composition of cerebrosides from sea cucumber *Stichopus variegatus* exhibited cytotoxicity against human colon cancer cell and induced apoptosis. They are major constituents of c-17, c-19 alkyl chain and 1-3 double bonds (Sugwara *et al*. 2006). Triterpene glycosides are the predominant secondary metabolites of the sea cucumber *Hemoiedema spectabilis* which exhibited wide spectra of biological activities, including antifungal, cytotoxic, hemolytic, cytostatic and immunomodulatory functions Chludil *et al*. (2002). A new lanostane-type triterpene glycoside, impatienside A and bivittoside D were isolated from the sea cucumber *Holothuria impatiens* Sun et al. (2007). The potential angiogenesis inhibitors, a novel sulfated saponin philinopside A, isolated from the sea cucumber *Pentacta quandrangulari*, possessed dual antiangiogenic and antitumour effects (Tong et al. 2005).

Fuscocineroside C bioactive compound obtained from sea cucumber *Holothuria fuscocinerea* a triterpene glycoside showed cytotoxic nature against human cancer cells Zhang et al. (2006). Hillaside C a triterpene derived from sea cucumber *Holothuria hilla* inhibited the growth of human leukemia, breast and colon cancer cells *in vitro* in a dose and time-dependent manner by a mechanism that required induction of apoptosis and the concomitant reduction of the apoptosis-suppressing protein Bcl-effect Wu et *al.* (2006). Intercedenside D–I iso lated a cytotoxic triterpene glycoside from the sea cucumber *Mensamaria intercedens* a marine natural product inhibited proliferation of several human cancer cell lines Zou et al. (2005). Steroid glycosides are a class of wide-spread natural products having marine origins. Spirostan and furostan steroid saponins, pregnane glycosides have a potential to be used as cancer therapies. Structurally, these glycosides exhibit a moderate cytotoxicity against human leukemia cell lines (Prassas and Diamandis 2008). Linhardt et al. (1990) found that low molecular weight sulphated polysaccharides are noted from sea cucumbers with efficient anticoagulant activities and several pharmacological properties. The chondroiton and glucosamine components of holothuria were reported to be important cartilage building blocks and other bioactivities including anti-inflammatory and anti tumor activity properties (Herecia and Ubeda 1998).The extract LPS obtained from *Stichopus japonicus* induced inflammatory response via blocks the MAPK signaling pathway in murine macrophages, showed *in vitro* with anti-inflammatory potential Himayaa et al. (2010). The sea cucumber *Telenata ananas* derived bioactive compounds were reported to act as the chemokine receptor subtype-5 (CCR5) with possible anti-HIV activity Hegde et al. (2002). Potential use of sea cucumber *S. liouvillei* isolated compound chondroitin sulfate (the polysaccharides) are reported to exhibit antiviral activity to inhibit human immunodeficiency virus (HIV) infection (Chen 2003). Considering this as an evidence in the present study, attempts were made to find out the bioactive compounds from marine invertebrates such as the Holothuria.

## Materials and methods

### Sample collection and extract preparation

*Holothuria atra* specimens with a size range of 10 to 30 cm in length and 30 to 180 g weight were collected from fishing nets operated off Kanyakumari (8° 03′ and 8° 35′ of the north latitudes and 77° 15′ and 77° 36′ of the east longitudes) in the Indian Ocean. Immediately upon collection, they were dissected to remove the internal organs and packed using ice prior and kept at –80°C or extraction. The skin portion was peeled off and stored in methanol in separate containers. The biologically active compounds were extracted as a function of their polarity using water and organic solvents. About 200 g of frozen samples were homogenized with deionized water and methanol. The mixture was continuously stirred in the dark at 4°C for 24 h. Then it was centrifuged at 5000 rpm for 15 min. The supernatant was collected and filtrated. The collected organic extracts were freeze-dried and kept at -80°C, while the insoluble solid materials were re-extracted with methanol (100%) (Chen 2003).

### MTT assay using Hela cell lines and MCF-7 cancer cell lines

The cells were preincubated at a concentration of 1 × 10^6^ cells/ml in culture medium for 3 h at 37°C and 6.5% CO_2_. Then, the cells were seeded at a concentration of 5 × 10 ^4^ cells/well in 100 μl culture medium and at various concentrations of extracts (dissolved in 2% DMSO dimethylsulphoxide solution) into microplates (tissue culture grade, 96 wells, flat bottom) and incubated for 24h at 37°C and 6.5% CO 2. Then, 10 μl MTT labelling mixture was added and incubated for 4 h at 37°C and 6.5% CO 2. Each experiment was conducted as triplicates sets. Then 100 μl of solub ilization solution was added into each well and incubated for overnight. The spectrophotometric absorbance of the samples was measured using a microplate (ELISA) reader. The wavelength to measure absorbance of the formazan product in 570 nm according to the filters available for the ELISA reader was used. The reference wavelength was more than 650 nm. IC_50_ values were calculated Percentage inhibition of novel compounds against all cell lines was calculated using the following formula:


### Trypan blue dye exclusion test

Being an essential dye, Tryphan blue was used in estimating the number of viable cells present in a population. The culture sample was mixed to resuspend cells. 20 μl of cell culture sample was taken and filled into sterile microfuge tube. To this 20 μl of 0.4% Trypan blue solution was added and mixed well by gently aspirating and dispensing the solution with the help of micropipette. The coverslip was fixed on the centre top of the hemocytometer. To the 10 μl mixture of the cell culture t he Trypan Blue mixture taken from the microfuge tube was added and kept in the hemocytometer assembly on microscope stage using 100 X magnification. The number of live and dead cells were recorded.


### Plaque reduction assay

Vero monolayer cells grown in 24 well tissue culture plates were infected with HSV -1 and HSV-2. Virus dilutions were made from 10^1^ to 10^7^ using 0.1 ml of viral suspension. Virus adsorption was carried out for 1h at 37°C in the presence of test extract. Virus dilutions were prepared in Eagles minimum essential medium. Prior to incubation, an overlay medium comprising of 0.8% carboxy methyl cellulose with 2% FBS was added. It was done to avoid formation of secondary plaques. Infected cell cultures were incubated at 37°C at 5.0% CO_2_ incubator for 2 to 3 days. The infected cells were stained and observed for plaque reduction. The infectivity titers were expressed as the number of plaque forming units per ml (pfu ml^−1^). After incubation, cultures were stained with 1% (w/v) crystal violet solution. The plaques were counted by visual examination and the percentage of plaque inhibition was calculated. The Pfu = Plaque number × reciprocal of dilution × reciprocal of volume in ml.

The antiviral activity was defined as the percentage of plaque inhibition as follows:


### Column chromatography

Silica (230-400 mesh) gel slurry was prepared using methanol. The column was packed with silica gel. After washing the column with same solvents, the sample (3 to 5 ml) was poured and then eluted with methanol: water on different percentages (10 to 100% methanol). The active fractions were collected and used for NMR analysis.

### Antibacterial activity

Disc diffusion method was employed to test the active fractions of *H. atra* obtained from the column chromatography Bauer et al. (1966). Antibacterial activity was determined using Muller Hinton agar (Hi Media).The bacterial cultures were obtained from the Microbial type culture collection and gene bank (MTCC), Institute for Microbial Technology, Chandigarh, India. They were *Staphylococcus aureus* MTCC 737, *E.coli* MTCC 443, *Klebsiella pneumonia* MTCC 109, *Listeria monocytogenes* MTCC 1143, and *Serratia liquefaciens* MTCC 3039. The plates were aseptically streaked with the test microorganism using a sterile swab and allowed to dry for a few minutes. Sterilized filter paper discs (Whatman no.1; 6 mm diameter) were used. The fractions were collected from 10 to 100% levels of methanolic extracts and were evaluated at 100 μl concentration. The plates were then incubated for 24 h at 37°C. Controls were blank discs impregnated with solvent. The diameter of the inhibition zone formed around the disc was measured.

### GC-MS analysis

The methanol extract of the sea cucumber *H. atra* was analyzed by GC-MS (Make: Fisons GC8000 series and MS: md800). The GC column dimension was: 30 mm, 0.25 mm, 0.5 mm AB-35MS fused silica capillary column. The GC conditions were as follows: injector temperature 250°C column temp isothermal at 100°C then programmed to rise up to 250°C at 6°C/min and held at this temperature for 10 minutes. The ion source temperature was 200°C and the interface temperature was 250°C. Helium gas was engaged for carrier gas at the rate of 1ml/min. Spectra was obtained in the EI mode with 70eV ionization energy. The compounds were identified by comparison with the standards. If not available, the mass spectra was matched with inbuilt library like wileys, NIST (Stonik et al. 1998).

### NMR analysis

The active fractions obtained from column chromatography were analysed for Nuclear magnetic resonance spectroscopy (NMR) analysis. Optical rotations were measured on a Perkin- Elmer Model 341 LC polarimeter. ^1^H NMR and ^13^C NMR experiments were performed on Bruker Unity 400 and 600 MHz spectrometers. NMR spectra were referenced to the CD3OD solvent signals at δ 3.30 (1H) and 49.00 (13C), respectively. The spectra were obtained using the standard Bruker software. The samples were dissolved in different solvents (i.e. DMSO-*d*6, CDCl3, and CD3OD), the choice of which was dependent on the solubility of the samples. The observed chemical shift (δ) values were given in ppm and the coupling constants (*J*) in Hz.

## Results and discussion

**Figure 1:**
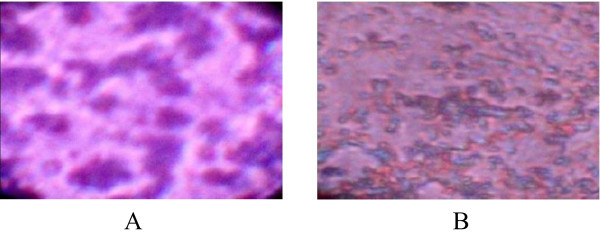
**Cytotoxic effects of** ***H.atra*** **extracts on A) Hela and B) MCF-7 cell lines.**

The results in Table 1 show that the amount of sea cucumber *H. atra* extract required to inhibit 50% of the antitumor activity against the Hela cell lines in 96 well plates could be determined. Antitumor activity was measured by using the IC_50_ values. The anti proliferative effect (IC_50_ value) exhibited by the *Holothuria atra* was 468.0 against the cervical cancer cell line (Hela). The cell inhibition was determined from the extract concentration ranged from 0.078 mg/ml to 10 mg/ml. The absorbance values were measured at 570 nm. Percentage of growth inhibition was identified at different concentration of the extracts. The gradual decrease in absorbance values showed increase in inhibition effect of the extracts against the Hela cell lines. The findings suggest that *H. atra* showed cell inhibition to the tune of 90% to the maximum in Hela cells and 75% of cell inhibition in MCF-7 cells. Five cerebrosides, PA-0-1, PA-0-5, PA-2-5, PA-2-6 and CE-2c were reported from the Japanese sea cucumber *Pentacta australis* Higuchi et al. (1994). A ganglioside molecular species SJG-1, isolated from the sea cucumber *Stichopus japonicus.* SJG-1 possessed a sialic acid, nonhydroxy fatty acids and phytosphingosine-type long chain bases as major ceramide components. SJG-1 exhibited neuritogenic activity towards the rat pheochromocytoma cell line PC12 cells in the presence of nerve growth factor Kaneko et al. (1999). Additionally, a cerebroside isolated from the sea cucumber *Stichopus japonicas* showed effective antitumor activity (Hayashi et al. 1990). The data presented in the Table 2 suggested the antitumor activity of the methanol extracts of *H. atra* against the breast cancer cell lines MCF-7 in 96 well microtitre plates. The susceptibility of cells to the extract exposure was characterized by IC_50_ values. Results indicated that the anti proliferative effect increased with the increase in concentration of the extracts. The IC_50_ value for the sea cucumber *Holothuria atra* was 352.0 A decrease in number of viable cells with the increase in concentration of the extracts was noted. From Table 3 the results of cell counting and viability of cells using tryphan blue staining were indicators for the influence of extracts. The percentage cell inhibition of Hela was 81.81% and MCF-7was 72%. The cell proliferation and inhibition measurment of the sea cucumber *Holothuria atra* showed that it can be developed as an antitumor agent. The cytotoxic effects of extracts against the Hela and MCF-7 cell lines are observed through the inverted microscope (Figure 1). Promising *in vitro* cytotoxic compounds such as the Calcigeroside B, C1 and C2 identified from the holothurians included triterpene glycosides. They showed antiproliferative action against the human and murine tumour cell lines (Alejandro and Gustafson 2003). In the present study, the tumor growth was inhibited by *Holothuria atra* extract and dosage was an important criteria which influenced the efficacy of the cell death in Hela, and MCF-7 cell lines. These suggest the possibility of apoptotic cell death through the activation of Bax a proapoptotic protein.

**Table 1:** **Cytotoxicity analysis of** ***Holothuria atra*** **extracts against Hela cell lines using MTT assay**

Concentration (mg/ml)	Absorbance (570 nm)	% inhibition	IC _50_
0.078	0.125	40.56	468.0
0.156	0.103	52.12	
0.3125	0.10	60.42	
0.625	0.093	68.30	
1.25	0.085	75.34	
2.5	0.070	83.26	
5	0.069	88.32	
10	0.032	90.56	
Cell control	0.45	100

**Table 2:** **Cytotoxicity analysis of** ***Holothuria atra*** **extracts against MCF-7 cell lines using MTT assay**

Concentration (mg/ml)	Absorbance (570 nm)	% inhibition	IC _50_
0.078	0.120	30.22	352.0
0.156	0.102	35.64	
0.3125	0.10	48.72	
0.625	0.088	55.60	
1.25	0.085	60.62	
2.5	0.075	65.20	
5	0.062	70.22	
10	0.052	75.60	
Cell control	0.45	100

**Table 3:** **Percentage cell inhibition and characterization of cell line exposed to** ***Holothuria atra*** **using tryphan blue**

Cell line	% cell inhibition	Dead cell count	Total cell count	pH
HeLa	81.81	1.80 × 10^5^	2.20 × 10 ^5^	6.9
MCF-7	72.72	1.76 × 10^5^	2.42 × 10 ^5^	7.2

Angiogenesis inhibitors and aromatase inhibitors present in sea cucumbers play a major role in reducing the growth of breast cancer and prostate cancers, especially the solid tumors. Research results showed that angiogenesis inhibitors effectively block the growth of tumors by cutting off their nutrient and blood supply. The mechanism by which they block tumor growth is driven by the inhibition of receptor tyrosine kinases (RTKs) that are over expressed by cancer cells (Chi 2006). Using MTT assay it could be inferred that *H. atra* extracts can block the growth of breast cancer cells (MCF-7) by inducing apoptosis. In *H. atra* extract there is a possibility of blocking the receptors such as the tyrosine kinases (RTKs) in cancer cells of Hela and MCF-7. The methanol extracts of *Holothuria atra* showed maximum inhibition of antitumor cells and it was observed by cell inhibition using tryphan blue.

**Figure 2:**
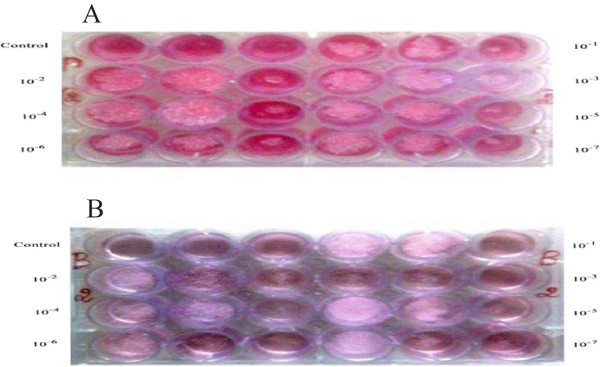
***In vitro*** **antiviral activity of** ***Holothuria atra*** **extracts against A) HSV-1and B) HSV-2 using plaque reduction assay.**

The concentration of extracts for *H. atra* was from 10 μg/ml to 70 μg/ml, respectively. The tested viruses were affected with the increase in concentration of extracts. The *H. atra* exhibited significant antiviral activity, and suggested the potential role of extracts. The effect of inhibition in plaque formation was evaluated based on the 101 to 107 dilutions of HSV-1. and HSV-2. In *H. atra* the highest plaque inhibition rate was as at 75% with 2.4 × 10^3^ pfu ml^−1^. Less inhibition rate was observed at 33% for 6.0 × 10^9^ pfu ml^−1^. The results of (Figure 2) suggest the effects of *H. atra* extracts on the inhibition of virus replication after attachment of HSV-1and 2 on Vero cells. In *H. atra,* the plaque inhibition obtained was high at 74% with 2.3 × 10^9^ pfu ml^−1^ whereas less effect was seen in *H .atra* was 27% with 6.4 × 10^3^ pfu ml^−1^ units (Table 4). Saponins the secondary metabolites which are triterpene glycosides present in sea cucumbers like *H. forskali* are reported to have antiviral property by *in vitro* and *in vivo* methods (Kerr and Chen 1995). It was observed that the *H. atra* extracts exhibited antiviral activity on plaque reduction assay in which maximum effect was seen against the HSV-1 to the tune of 74%. Thus *Holothuria atra* extracts have the ability to arrest the multiplication of virus and suppress its growth by influencing the growth factors. This could have resulted in appearance plaques in the plaque reduction assay. Bioactive peptides and hemolytic lectins have been reported from sea cucumber as a source of antiviral activity. Among Holothuroidea genera, *Cucumaria echinata* and *C. frondosa* contained lectin and peptide, respectively. They have been found in the body wall mucus Hisamatsu et al. (2008). Fucoidans, polysaccharides containing substantial percentages of L-fucose and sulfate ester groups are generally considered as constituents of sea cucumbers. Fucoidan can inhibit the development of cytopathic effect (CPE) and protect cultural cells from infection caused by viruses (Hemmingson et al. 2006). This can induce the antiviral effects against the Herpes simplex viruses.

**Table 4:** **Inhibitory action of** ***Holothuria atra*** **extracts against HSV strains (- ?)**

Dilution of virus	Concentration of ***H. atra***extracts	Plaque forming units pfu/ml		% Plaque inhibition [HSV-1 and HSV-2]	
		HSV-1	HSV-2	H. atra	
Control	Nil	9.2 × 10^1^	8.7 × 10 ^1^	-	-
10^−1^	10 μg	6.0 × 10^9^	6.4 × 10 ^3^	33	27
10^−2^	20 μg	5.6 × 10 ^8^	5.6 × 10 ^4^	40	36
10^−3^	30 μg	5.2 × 10^7^	4.7 × 10^5^	44	46
10^−4^	40 μg	4.4 × 10 ^6^	4.3 × 10 ^6^	53	51
10^−5^	50 μg	3.9 × 10 ^5^	2.3 × 10^9^	58	60
10^−6^	60 μg	3.3 × 10 ^4^	2.8 × 10 ^8^	65	68

**Figure 3:**
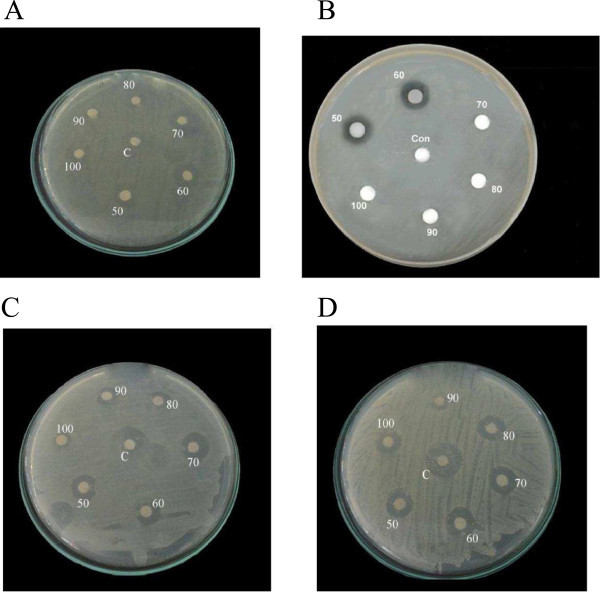
**Anti bacterial activity of the column purified fractions of sea cucumber,** ***Holothuria atra*** **against A)** ***Serratia liquefaciens*** **B)** ***Escherichia coli*** **C)** ***Klebsiella pneumoniae*** **D)** ***Staphylococcus aureus.***

**Figure 4:**
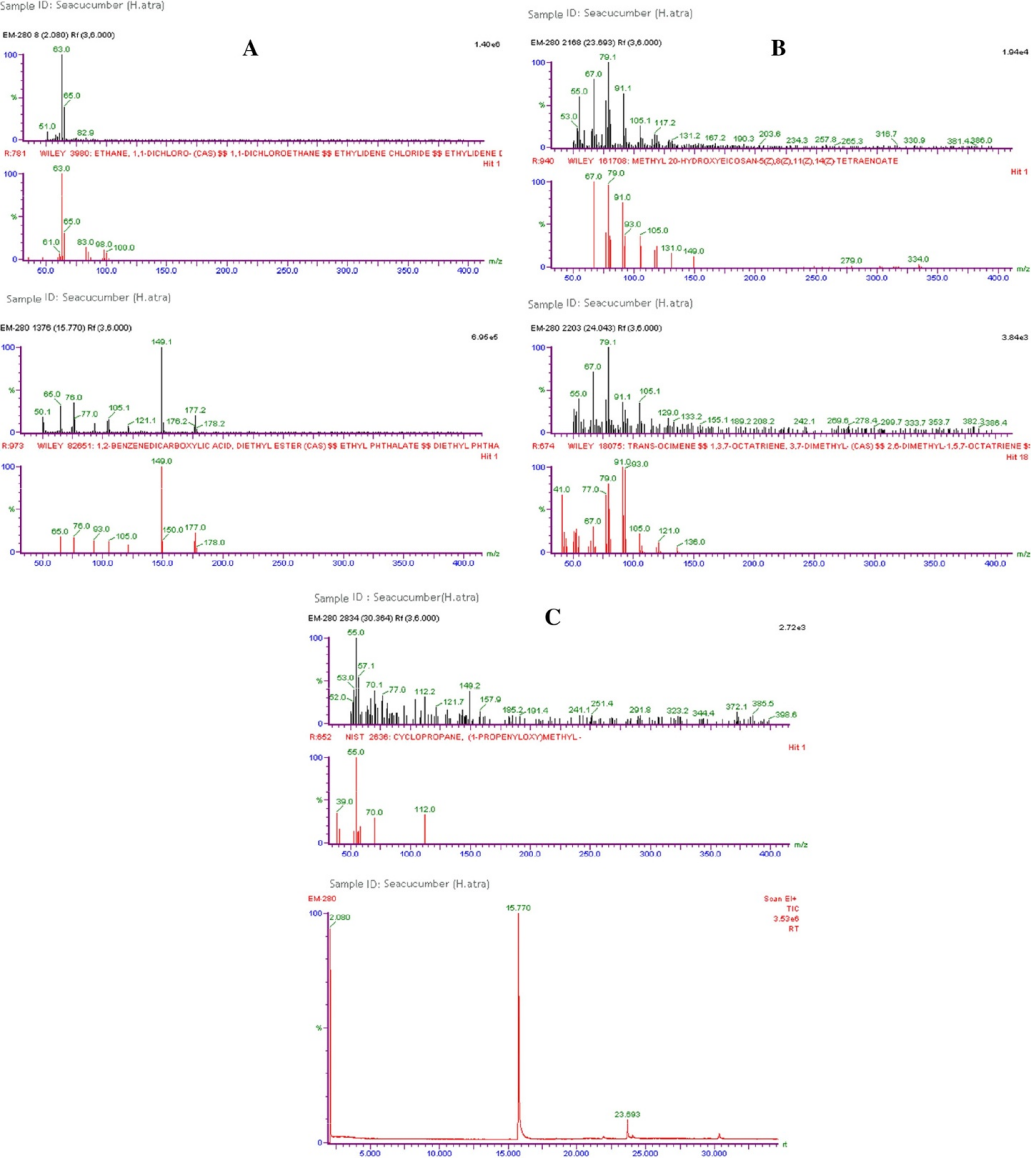
**GC-MS Analysis of the methanolic extracts of the sea cucumber** ***Holothuria atra.***

Figure 3 shows the antibacterial activity for the active fractions obtained from column chromatography of *H. atra* against various Gram positive and Gram negative bacteria viz., *Klebsiella pneumonia*, *Serratia liquefaciens*, *Staphylococcus aureus*, *Listeria monocytogenes,* and *Escherichia coli*. The fractions collected from 50 to 100% showed maximum effect whereas the fractions from 10 to 40% did not show any activity. In *H. atra* the *E. coli* had less effect at 50 and 60% of fractions which showed the zone diameter of 2 and 3mm range. Figure 4 shows the GC-MS graphical representation of the extracts of sea cucumber. The interpretation on mass spectrum GC-MS was carried out and the spectra of the unknown component were compared to the spectrum of the known components stored in the NIST library. The name, molecular weight of the components was ascertained. A total of 59 natural compounds were identified from the extracts of sea cucumber (*H. atra*)*.* The active principles with their retention time (RT), molecular formula, molecular weight (MW) were ascertained (Table 5).

**Table 5:** **Compounds identified in the crude methanolic extracts of sea cucumber (** ***Holothuria atra*** **) using GC-MS Analysis**

No	Name of the compound	Molecular formula	M.W
1	Ethane 11dichloro(CAS)11dichloro ethane.	C2H4Cl2	98
2	Carbamic acid,hydroxyl, ethyl ester	C3H7O3N	105
3	Ethane sulphonyl chloride,2 chloro	C2H4O2Cl2	162
4	Methyl 40-methyl-beta-D-xylophyanoside	C7H1405	178
5	Propylhexedrine	C10H21N	155
6	2-methyl-1,3-dithiacyclopentane	C4H8S2	120
7	Ethane dithioic acid	C2H4S2	92
8	4 ketopinelic	C7H10O5	174
9	2-ethylomethyl-1,3-dithiacyclopentane	C8H16S3	208
10	Carbomic chloride,methoxymethyl	C3H6O2NCl	123
11	Xylane,ethyldimethyl	C4H12SL	88
12	D-pseudo ephedrine or ephedrine or pholedrine	C10H15ON	165
13	Ethanol,1-methoxy-benzoate	C10H12O3	180
14	Methanomine hippurate artefact	H40	0
15	2,6,10,14 tetra methylpentadecane-2-ol	C1940O	284
16.	2,5,8-heptadecatrien-1-ol	C17H30O	250
17	Methyl 19-hydroxyeicosan-5(z),8(z),11(z),14(z)-tetrae.	C21H34O3	334
18	3,4epoxy-6,9-octadecadiene	C18H32O	264
19	(14z,17z)-3,20-dibromo-21-ethyl-2,6-epoxy-1-oxacyclo	C22HO3B12	496
20	(-)-elema-1,3,11(13)-trien-12-ol or Beta –costol	C15H24O	220
21	Phenylpropanolamine	C9H13ON	187
22	3,3-dibromotricyclo(2,4) undecane	C11H16B12	306
23	Transcaryophyllene or Alpha far nesene or epsilon cadinene.	C15H24	204
24	2,3-2poxy-5,8-hectadecadien-1-ol	C17H30O2	266
25.	(+-)-3-methylidenetricyclo(2,9)undecan-8-one	C12H16O	176
26	7-methylbicyclo oct-7-ene	C9H14	122
27	Methylareachidonate 5,8,11,14 eicosatetraenoic	C21H34O2	318
28	2 –naphthalenemethanol-decahydro-5-methylene-8	C 14H22O	206
29	Tricyclo decanedimethanol	C12H20O2	196
30	2-3-29Methoxycarbonyl) ethyl bicyclo 1-1-1pent-1	C13H18O4	238
31	Transcaryophyllene.	C15H24	204
32	2xo,2endo:3endo,3exo-bis (epoxymethane) bicyclo	C9H12O2	152
33	2-pyrrolidinnarbocylic acid.5-methyl,phenylne	C13H17O2N	219
34	Cyclopropane,1-etenyl-2-hexenyl,1apha,1 e Bet	C11H18	150
35	Propene,3-phenyl-3-ol	C9H10O	134
36	1-bromo-12cyclohexylidene-3-buen-2ol-$$3-buten	C10H15Obr	230
37	2-4 nonadien-6-yn -1-0l,(e,e)	C9H12O	136
38	Tricyclo3,2,1,02,4 octane 8-one,3,3-dimethyl,(1 alp)	C10H14O	150
39	Trans-ocimene 1,3,7-octatriene,3,7- dimethyl	C10H16	136
40	Cycloprpane,{(1-propenyloxy) methyl}- (CAS) CY	C7H12O	112
41	3T-Bromo-1-ethynyl-1c,2r-cyclohexanediol	C8H11O2Br	218
42	Didodecyl phthalate	C32H54O4	502
43	Phendimetrazine	C12H17ON	191
44	Methyl 2-ethylhexyl phthalate	C17H24O4	292
45	Di 2 ethylhexyl phthalate	C24H38O4	390
46	1,2-benxenedicarboxylic acid, dioctyl ester (cas)	C22H34O4	362
47	5,8-methanospiro(4,5) deccane-1,4 dione	C11H14O2	178
48	3-octyl acetate	C10H20O2	172
49	Bicyclo[3.2.1]octan-2-one, 1-(1-propenyl)- a(cas) 1-p	C11H16O	164
50	Ethylphenyl-n-ethylamine	C10H15N	177
51	Phthalic acid, didecyl ister 1,2-benzenedicarbo	C28H46O4	446
52	Bupranolol (betadrenol)	C13H2102N	271
53	Ethosuxinide (sematin)	C7H11O2N	141
54	1,2-Benzenedicarbozylicacid,diethy ester (CAS)	C12H14O4	222
55	2-acetylbenzoic acid	C18H26O5	164
56	1,2-Benzenedicarboxylic acid dipropyl ester(CAS)	C14H18O4	250
57	Nitrofurantion	C8H6O5N4	238
58	Benzoic acid-4 forml	C8H6O3	150
59	1,2-Benzenedicarbozylicacid dibutyl ester(CAS)	C16H22O4	278

Fucoidan of sea cucumber *Laminaria japonica* has anti RNA and DNA virus functions. The antivirus effects of fucoidan on infection was against poliovirus III, adenovirus III, ECHO6 virus, coxsackie B3 virus and coxsackie A16. Fucoidan inhibited the development of cytopathic effect (CPE) and protected the cultural cells from infection caused by the viruses Li et al. (1995). Sulfated polysaccharides from sea cucumbers such as the *Cucumaria japonica, Holothuria impatiens* are reported to exhibit antiviral activity. Based on this fact, Japanese scientists have patented their scientific findings regarding the potential use of sea cucumber chondroitin sulfate to inhibit human immunodeficiency virus (HIV) infection Beutler et al. (1993). Triterpene glycosides, namely holothurinosides A, B, C and D as well as desholothurin A from sea cucumber (*Holothuria forskali*), have considerable antitumour activity against P388 cell lines. The saponins isolated from the aqueous and methanolic extract of sea cucumber (*Holothuria forskali*) have showed considerable antiviral activities Mulloy et al. (2000). Considering these as well as the results of present investigations, the methanolic extracts of *Holothuria atra* could form effective antitumour and antiviral agents. Previous work showed that sulphated polysaccharides such as glycosaminoglycans an inhibitor of human immunodeficiency virus binds to T lymphocytes and showed antiviral activity Toido et al. (2003). In low concentrations, the extract showed potent inhibitory effect towards Herpes simplex virus and thus has got significant drug value. The antiviral activity using the sea cucumber *Ludwigothurea grisea* and *Thelenota ananas* derived fucosylated chondroitin sulfates (FCS), was recognized as the sulfated polysaccharides. It inhibited human immunodeficiency virus (HIV) infection Mc Clure et al. (1992). The present work suggested that *Holothuria atra* extracts showed virucidal action through reduction in number of plaques formed during plaque reduction assay against the HSV-1 and HSV-2. The strong growth inhibitory activity found in the extract of *Holothuria atra* might be the source for the development of antiherpetic compound.

**Figure 5:**
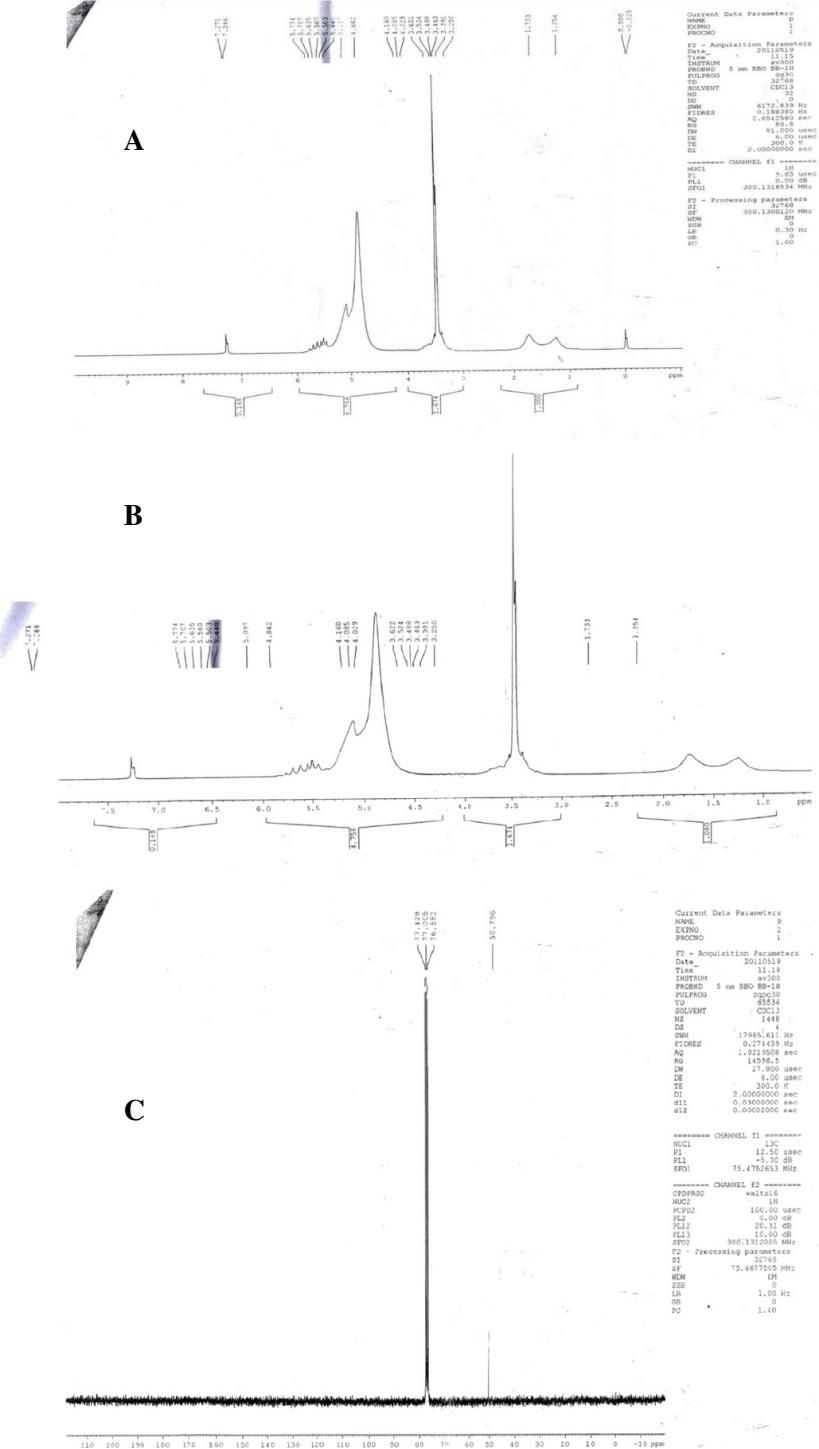
**NMR analysis of active fractions obtained from Column chromatography (A)** ^**1**^ **H NMR Spectrum of** ***Holothuria atra*** **(B)** ^**1**^ **H NMR Spectrum of** ***Holothuria atra*** **(C)** ^**13**^ **C NMR Spectrum of** ***Holothuria atra.***

The structure of bioactive compounds was elucidated by using NMR spectra from the active fractions. In addition, the methyl groups were observed in the ^1^H NMR spectra including singlets and doublets which were integrated relatively for olefinic proton at δ position. The 13C NMR spectrum showed the presence of a carbon–carb on double and indicated the presence of two conjugated carbonyls. It also showed the appearance of two carbon signals. Figure 5 represent the 1H and 13C NMR data of *Holothuria* atra. Some of the bioactive compounds identified with their structures are given below. Sea cucumber derived fucosylated chondroitin sulfates (FCS) which inhibited the growth of human immunodeficiency virus and also acted as a cytotoxic agent was initially obtained from *Stichopus badionotus* Kaswandi et al. (2004). It could be predicted that high molecular weight compounds present in *Holothuria atra* detected by NMR analysis could form a potent antiviral sources (Additional file 1).

## Conclusions

The *H. atra* extract had various compounds such as the flavonoids, phenolic components, terpenoids, saponins, alkaloids etc. The GC-MS analysis revealed the presence of 59 compounds. It was found that *H. atra* extracts showed anti proliferative activities against the Hela and MCF-7 cell lines. Similarly the inhibitory action of extracts were found against the HSV-1 and HSV-2 strains was analyzed by plaque reduction assay. From NMR analysis the structural elucidation of the active compounds were studied. These results will direct future efforts to optimize the anti proliferative activity of these bioactive compounds.

## Electronic supplementary material

Additional file 1: **Structures ofbioactive compounds as based on NMR specrum [Figure** 5 **].** (DOC 378 KB)
