# Supplementary material for: Bioactive compounds from Holothuria atra of Indian ocean
Source: Springerplus. 2014 Nov 14;3:673. doi: 10.1186/2193-1801-3-673 (PMC4240768; doi:10.1186/2193-1801-3-673)
Supplement: Supplementary file 1 — Additional file 1: Structures ofbioactive compounds as based on NMR specrum [Figure 5 ]. (DOC 378 KB) [file 40064_2014_1371_MOESM1_ESM.doc]

**Structures ofbioactive compounds as based on NMR specrum [Fig 5]**


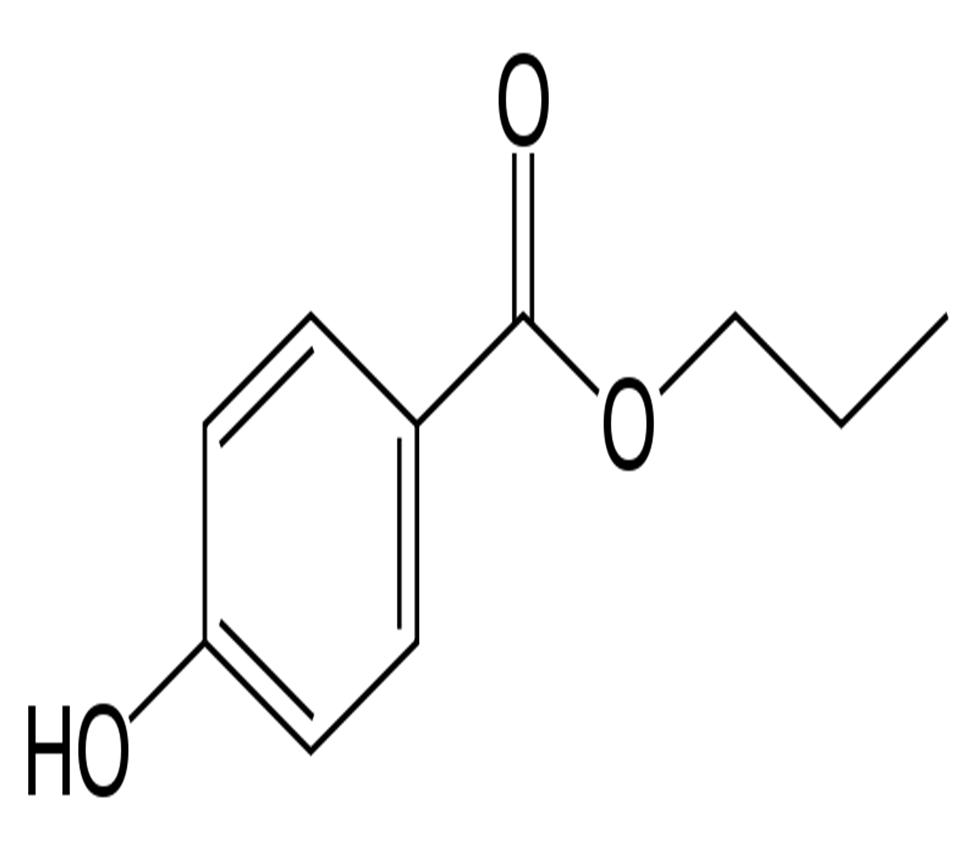

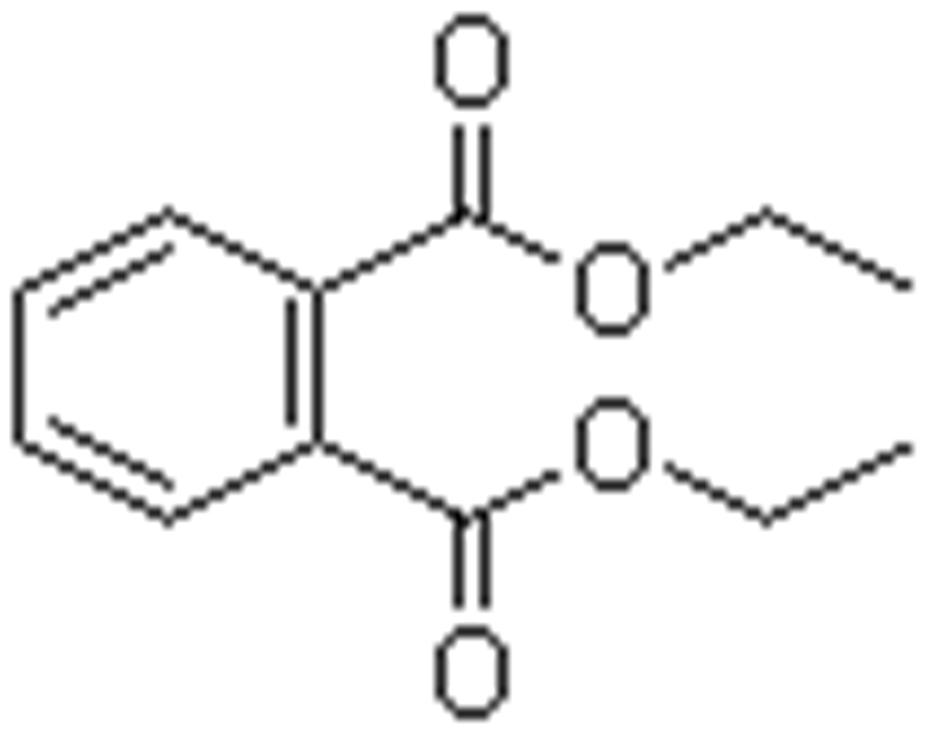


1)1,2-Benzenedicarbozylicacid,diethylester 2) Propylparaben


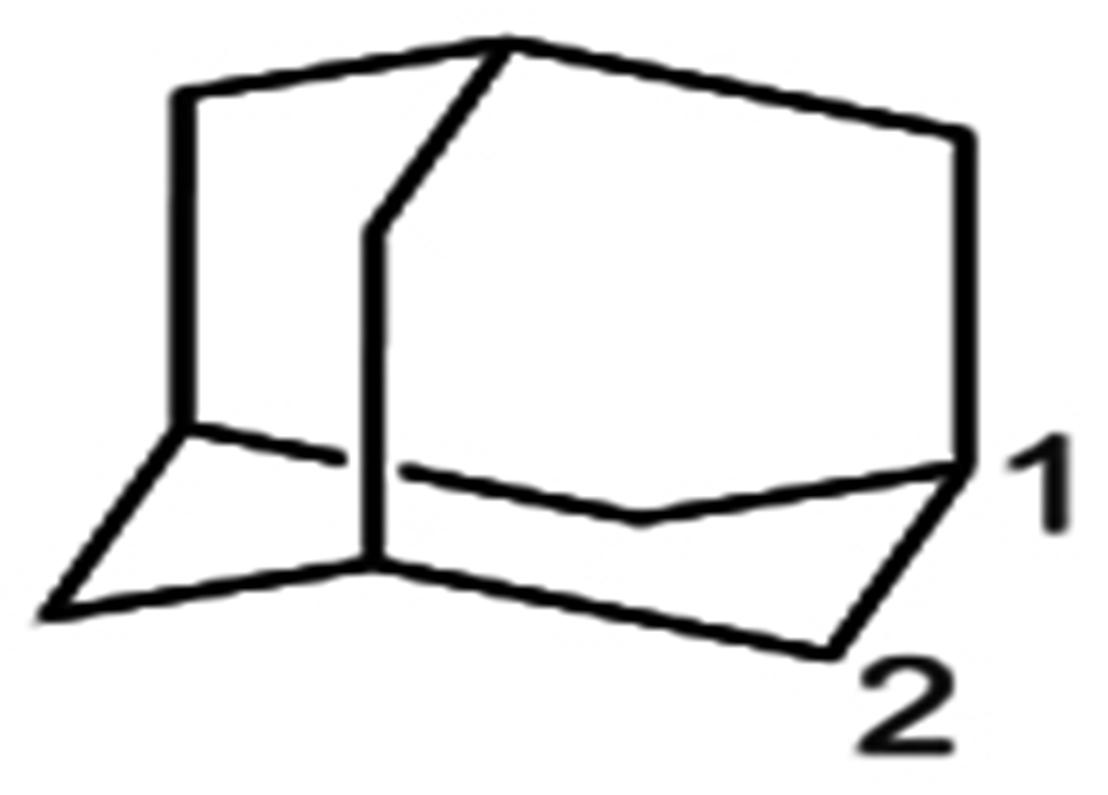

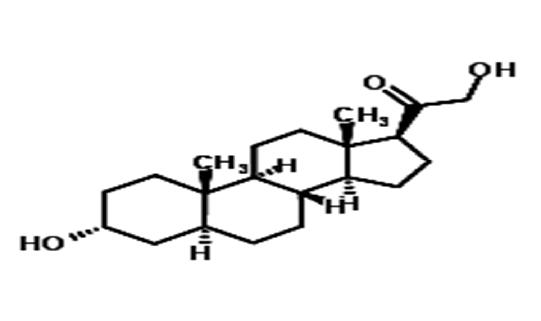


3) Tetrahydrodeoxycorticosterone 4) Adamantane


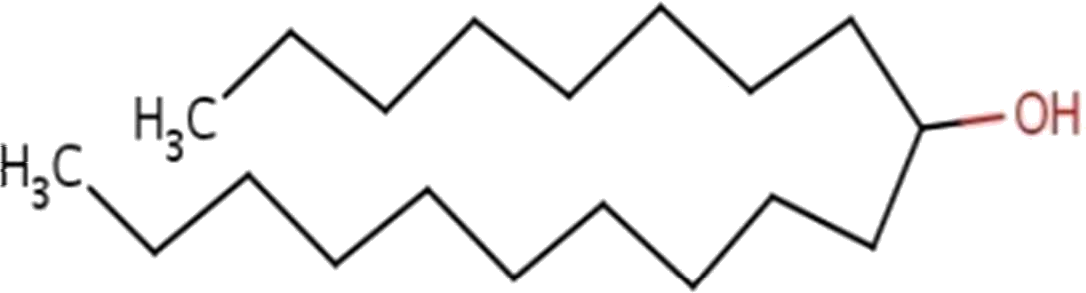

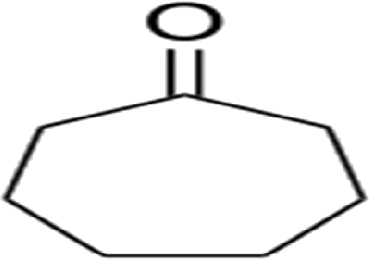


5) Cycloheptanone 6) N-(Trifluoroacetyl) Prolylmethamphetamine


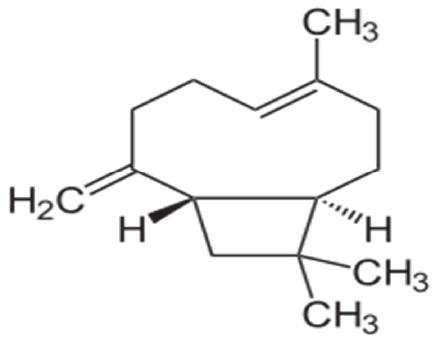


7)Transcaryophyllene (Sesquiterpene)
